# RAMTaB: Robust Alignment of Multi-Tag Bioimages

**DOI:** 10.1371/journal.pone.0030894

**Published:** 2012-02-08

**Authors:** Shan-e-Ahmed Raza, Ahmad Humayun, Sylvie Abouna, Tim W. Nattkemper, David B. A. Epstein, Michael Khan, Nasir M. Rajpoot

**Affiliations:** 1 Department of Computer Science, University of Warwick, Coventry, United Kingdom; 2 College of Computing, Georgia Institute of Technology, Atlanta, Georgia, United States of America; 3 School of Life Sciences, University of Warwick, Coventry, United Kingdom; 4 Biodata Mining Group, Bielefeld University, Bielefeld, Germany; 5 Department of Mathematics, University of Warwick, Coventry, United Kingdom; Aligarh Muslim University, India

## Abstract

**Background:**

In recent years, new microscopic imaging techniques have evolved to allow us to visualize several different proteins (or other biomolecules) in a visual field. Analysis of protein co-localization becomes viable because molecules can interact only when they are located close to each other. We present a novel approach to align images in a multi-tag fluorescence image stack. The proposed approach is applicable to multi-tag bioimaging systems which (a) acquire fluorescence images by sequential staining and (b) simultaneously capture a phase contrast image corresponding to each of the fluorescence images. To the best of our knowledge, there is no existing method in the literature, which addresses simultaneous registration of multi-tag bioimages and selection of the reference image in order to maximize the overall overlap between the images.

**Methodology/Principal Findings:**

We employ a block-based method for registration, which yields a confidence measure to indicate the accuracy of our registration results. We derive a shift metric in order to select the Reference Image with Maximal Overlap (RIMO), in turn minimizing the total amount of non-overlapping signal for a given number of tags. Experimental results show that the Robust Alignment of Multi-Tag Bioimages (RAMTaB) framework is robust to variations in contrast and illumination, yields sub-pixel accuracy, and successfully selects the reference image resulting in maximum overlap. The registration results are also shown to significantly improve any follow-up protein co-localization studies.

**Conclusions:**

For the discovery of protein complexes and of functional protein networks within a cell, alignment of the tag images in a multi-tag fluorescence image stack is a key pre-processing step. The proposed framework is shown to produce accurate alignment results on both real and synthetic data. Our future work will use the aligned multi-channel fluorescence image data for normal and diseased tissue specimens to analyze molecular co-expression patterns and functional protein networks.

## Introduction

Bioimage informatics is a rapidly growing branch of computational biology that has emerged in response to two major demands: increasing deployment of powerful new technologies for measuring molecular components (including genomics, transcriptomics, proteomics, metabolomics) and new biological knowledge (from the human genome project amongst others). Bioimage informatics is concerned with the processing, analysis, and management of images recorded for biological specimens mostly using microscopy techniques [1]–[3]. The ultimate objective is to localize molecular components in biological samples (ranging from cell cultures to tissue sections) in order to overcome one of the most important limitations of most traditional destructive ‘omics’ technologies, in which molecular phenotype is acquired at the expense of anatomical and cellular spatial information [4]–[6]. New techniques such as MALDI imaging [7] or Raman microscopy [8] record high dimensional images, organized as stacks of grey value images, encoding the co-location or interaction of a large number of molecules. Another group of new bioimaging approaches achieve this by using different fluorophores, multispectral analysis, or bleaching with only one fluorophore [9]–[12]. The resultant image data consist of a stack of *N* grey value images 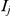 (*j* = 1, …,*N*) where each image shows the spatial distribution of one molecule. Due to these techniques becoming ubiquitous, new computational approaches are needed to process and visualize multivariate bioimages [13], [14].

Since most analytical approaches are based on processing *N* grey values 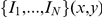 associated to a pixel (*x, y*) and searching the images for interesting patterns of co-location, for instance using clustering and dimension reduction [15], it is vital that all *N* images in a stack are aligned. Growth in molecular dimension is often accompanied with a growth in runtime of the imaging experiment. As a consequence, serious shifts can be observed between pairs of images in one stack, recorded for one field of view, making a direct analysis of the co-location signals meaningless. In experiments lasting for several hours, shifts can be caused by various external influences (mechanical perturbations, temperature changes, shift movements by the specimen due to repeated washes etc.)

In this work, we propose an efficient framework to align all images in an *N*-dimensional fluorescence image stack. For each biomolecular tag, our data is acquired as a pair of fluorescence and phase contrast images. The fluorescence images provide information about the relative expression level of respective tags in subcellular compartments and the phase contrast images are used for the purpose of alignment. Each fluorescence/phase contrast pair is assumed to be correctly aligned. The key idea is to determine the transformations necessary to align the phase contrast images and then apply these transformations to register the fluorescence images.

We use the toponome imaging system (TIS) [11], [12] which is an automated robotic microscopy system. It uses fluorescence imaging to locate tens to hundreds of different proteins or other biomolecules (in a cell or a tissue) by using fluorescence labelled antibodies, lectins or other specific ligands (referred to as tags, in general). One data set, a stack of grey value images, is recorded by performing *N* sequential cycles of fluorescent tagging, labeling and bleaching *in situ*. In each iterative step *j*, a fluorescence or tag image *F_j_* and a corresponding phase contrast image 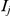 is recorded. So for each tag, e.g., an antibody against a specific protein or a dye such as DAPI that stains nuclei, we obtain fluorescence and phase contrast images. The aligned fluorescence images can then be further analyzed to determine biological properties.

**Figure 1:**
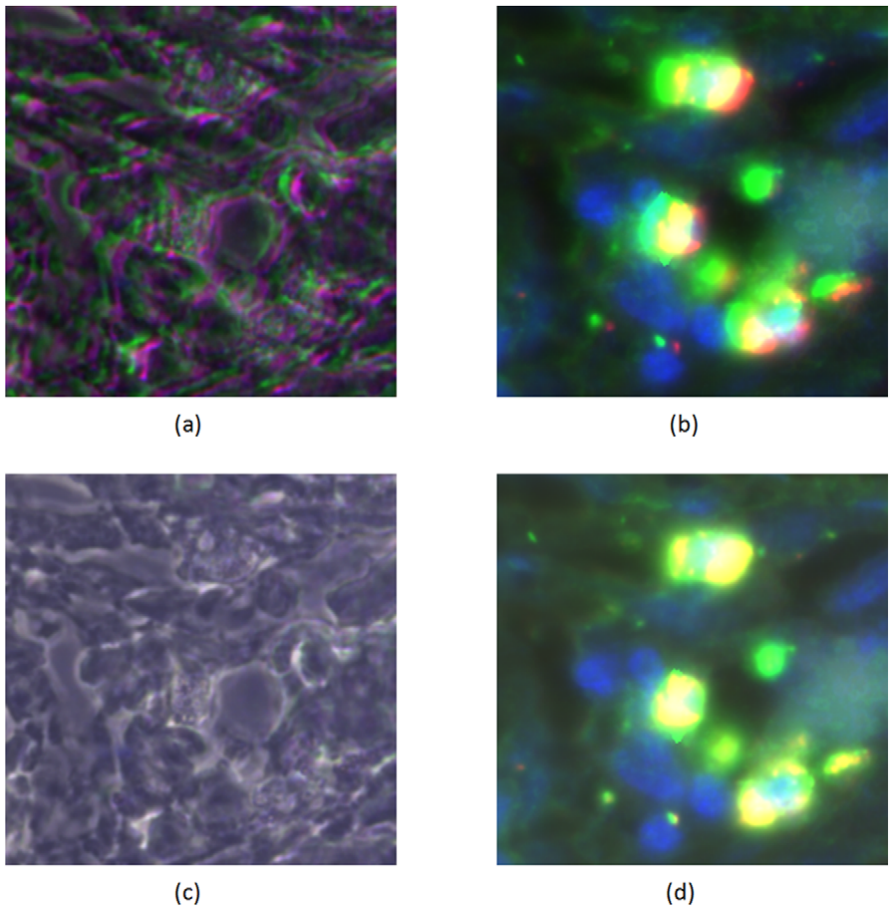
The RGB composite image before and after applying the RAMTaB: R,G and B channels belong to CD57, CD166 and DAPI tags respectively. (a) and (b) respectively show composite image formed by using phase images *I_j_* and fluorescence images *F_j_*, before alignment, (c) and (d) show the RGB composite images after the images were aligned using the proposed framework. The red color fringes in (a) show the degree of misalignment among the three tags. These color fringes have been replaced by white and grey regions in (c) after alignment.

We did not observe any significant misalignment between fluorescence and corresponding phase contrast images. However, we observed misalignment between phase contrast images for different antibody tags. The misalignment is manifested in terms of translational shifts. Other forms of misalignment, such as rotation, do not appear in our context, and we assume that all alignment transformations are translations. Figure 1 shows a misaligned composite RGB color image made up of CD57, CD166 and DAPI (DAPI binds to nuclei, while CD57 and CD166 are protein markers) tags displayed in red, green, and blue channels respectively.

If our work is adapted to fit other experimental situations, then it may become advisable to widen the class of alignment transformations, for example to include small rotations, but it appears extremely unlikely that we will ever need to do so in our situation.

**Figure 2:**
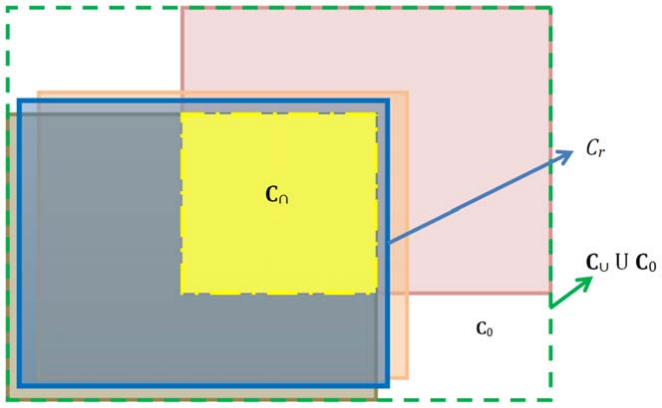
An illustration of mosaiced image containing all the aligned images.

The overall aim of this work is to compute transformation(in terms of translational shift) parameters for each tag image in a stack, such that a) the images are well aligned and b) the total number of non-overlapping pixels 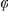 is minimized. This loss of information or total number of missing pixels 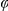 may vary from one reference image to another. Suppose we have a stack of *N* images 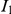, …, 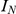, all of the same scene, though possibly not perfectly aligned with each other. We choose a reference image 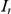, and then for each target image 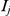 we find transformation 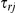 so that each point on 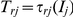 for all *j* = *1*,*2*, …, *N* corresponds to one and the same point in the tissue specimen being imaged. We will assume that the alignment transformations 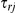 are always translations, which is a reasonable assumption in the situation to which we will apply our theory. The aligned images can be mosaiced and arranged in a larger frame of reference as shown by the green dashed line in Figure 2.

We address one of the several different ways (please see Appendix S1 for other possible options) in which registration results can be used for follow-up analysis. Let us fix *r* with 1≤*r≤N*, and let us restrict our analysis to the region 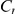. The signal from the *i*th image comes only from the region 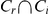. Then
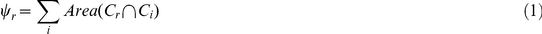
represents the sum of the areas overlapped by 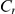 that provide meaningful signal. We find 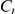 corresponding to 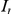 with 1≤*r≤N* such that 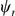 is maximal. Since the process of aligning images is usually referred to as registration in the domain of biomedical imaging, we will use this term in the remainder of this paper.

In this paper, we present a framework for robust registration of multi-tag fluorescence microscopy images. The method is based on three ideas. First, we use the phase contrast images 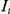, 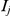 of two images *i* and *j* to compute the shift parameters for their corresponding fluorescence images *F_i_* and *F_j_*. Second, we propose a method that computes a confidence value for how well the registration algorithm performs on this particular pair of images (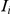,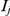). Third, we propose an efficient method for selecting the Reference Image with Maximal Overlap (RIMO) in order to maximize the total amount of data recordable within the co-ordinates of a single image. To the best of our knowledge, this problem has not been addressed in the microscopy imaging literature. A side benefit of automated RIMO selection is that the user (often a biologist) does not need to eyeball all images in a stack to select a reference image. The proposed RAMTaB framework for registration and selection of RIMO is not restricted to TIS image data and can also be applied to image stacks generated by other multi-tag bioimaging systems where both phase contrast and fluorescence images are acquired for every biomolecular tag.

### Related Work

There is a vast amount of literature on image registration; see for instance [16]–[19] for excellent surveys on registration of images. A large body of literature can also be found on multimodal image registration [20]–[23] in the domain of medical imaging. The problem of multi-channel image registration has also been associated in the literature with the inter-subject registration of 3D diffusion tensor magnetic resonance images; see for example [24]–[26].

In the case of multi-tag fluorescence microscopy, there is a dearth of literature on registration algorithms for such image data, primarily because imaging systems for such type of data have emerged only recently, although several researchers have proposed techniques for solving the somewhat related problem of automatic tracking of live cells by registering time consecutive frames; see for instance [27]–[29]. Wang *et al.* [30] proposed the M-FISH (Multiplex fluorescence *in situ* hybridization) algorithm for registration of multi-channel images in the context of cancer diagnosis and research on genetic disorders. Their algorithm searches for a transformation *T′* using mutual information to register the misaligned multi-channel FISH images. The authors selected DAPI as a reference image and did not address the problem of choosing the reference image to minimize *φ*. Kim *et al.* [31] have proposed registering multi-channel images using a three step procedure: 1) Gaussian filtering, 2) rigid registration and 3) non-rigid registration. For rigid registration, the authors minimized the mean-squared intensity error and for non-rigid registration, a variant of the Demons' algorithm [32] was used. They also presented two approaches for selecting the reference image. The first approach uses the first image in time as the reference image and all the images are registered to this reference frame. The second approach uses information from previous time steps in an incremental scheme. Can *et al.* [9] have used a mutual information based measure to register images from different histological imaging modalities. They mapped the multi-modal fluorescence images of same tissue stained with molecular biomarkers to the co-ordinate system of Hematoxylin and Eosin (H&E).

In this work, we specifically address the problem of multi-tag fluorescence microscopy image registration where multiple phase-and-fluorescence images of the same sample stained by different biological tags are obtained.

## Results

### Experiments on Synthetic Data

**Figure 3:**
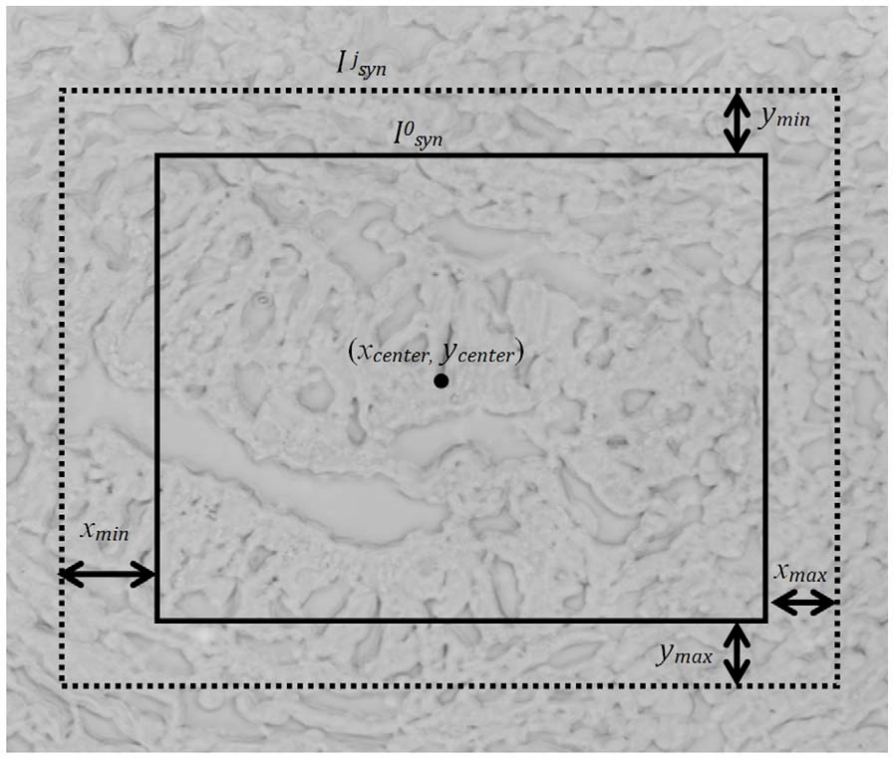
Construction of a synthetic data set I*_syn_* using a single tag image *I_sel_* with center coordinates (*x_center_*, *y_center_*) and uniformly distributed random shifts *x_n_*∈[*−x_min_,+x_max_*] and *y_n_*∈[*−y_min_,+y_max_*] in both directions. Intensity variations such as contrast stretching and Gaussian blurring are also introduced randomly in 50% of the images to mimick the random perturbations in pixel intensities during the image acquisition process (The image here has been inverted for visibility purpose.)

Synthetic data was generated by selecting a phase contrast image *I_sel_* from one of the TIS image stacks. Two random vectors *x*′ and *y′* of length 500 were drawn from a uniform distribution of real-valued numbers in the range [*−x_min_, +x_max_*] and [*−y_min_, +y_max_*] with *x_min_* = *x_max_* = *y_min_* = *y_max_*. = 10. Let (*x_center_*, *y_center_*) denote coordinates of the center of the selected image *I_sel_* and let 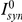 denote a cropped section of *I_sel_* with (*x_center_*, *y_center_*) as its center. A new set of center coordinates for the synthetic tag images is then calculated by adding 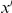 and 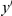 to (*x_center_*, *y_center_*) as follows,
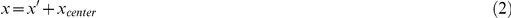


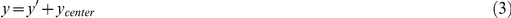
A synthetic stack of TIS images **I** *_syn_* = {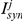}, where *j* = 1,2, …,500, is generated by taking cropped sections of *I_sel_* with (*x_j_*, *y_j_*) as their centers and having the same pixel resolution as 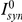. The amount of actual shift for the synthetic tag image 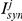, for all *j* from the original *reference* image 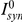 is given by the corresponding values (i.e., the *j*th elements) in *x* and *y*. Nearly a quarter of the synthetic tag images were randomly picked and a contrast change using gamma correction [33] with *γ* in the range 0.5 to 2 was applied to them. Another quarter of the synthetic images were randomly picked and Gaussian blurring with kernel bandwidth *σ* = 1 and a filter size of 5×5 pixels was applied to them. The remaining 50% of the images did not go through any intensity transformation, and were only translated by random shifts. So, a dataset consisting of randomly shifted images was generated, with contrast and smoothing artifacts added to half of them randomly. Figure 3 shows an illustration of how the synthetic data set is generated using a single phase contrast image from a TIS image stack as *I_sel_*.

An image was randomly selected from our artificial data set and shifts ***x_cal_*** and ***y_cal_*** were calculated using our registration algorithm. The mean difference between the actual and estimated shifts was calculated to be (0.1128, 0.1165) in the *x* and *y*-directions respectively. We can achieve more accurate results by using different values of *K*, *S* and 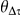 as shown in Table 1, but there is always a trade-off between time and accuracy. Using ***x_cal_*** and ***y_cal_*** the RIMO was calculated by using our algorithm. The RAMTaB successfully found the image which had minimum shift with respect to all of the other images of the stack, therefore providing experimental verification that the algorithm is capable of finding RIMO.

**Table 1:** Time required to register a stack of 26 images with two visual fields for different values of *K*, *S*, and *θ*_Δτ_ where *K* is the number of subimages used while calculating the translations, *S* is size of the subimages, and *θ*_Δτ_ is the threshold for |Δτ| between two consecutive iterations of the pattern search algorithm.

*K*	*S*	*θ*_Δ*τ*_	Time taken to register real data containing 26 tag images with two visual fields	Approximate error to register 500 images of synthetic data			
				Normal	Corrupted	Mean	Standard Deviation
9	100×100	0.01	9 min 48 sec	0.4229, 0.3230	0.4228, 0.3556	0.4229, 0.3393	1.7801, 1.3568
		0.001	20 min 55 sec	0.3101, 0.2225	0.3294, 0.2638	0.3198, 0.2432	1.7731, 1.3647
		0.0001	30 min 25 sec	0.3010, 0.2133	0.3233, 0.2585	0.3122, 0.2359	1.7744, 1.3667
9	200×200	0.01	22 min 15 sec	0.1107, 0.1161	0.1149, 0.1169	**0.1128, 0.1165**	**0.0662, 0.0652**
		0.001	51 min 38 sec	0.0135, 0.0142	0.0166, 0.0153	0.0151, 0.0148	0.0121, 0.0120
		0.0001	81 min 27 sec	0.0020, 0.0023	0.0072, 0.0067	0.0046, 0.0045	0.0095, 0.0099
6	300×300	0.01	42 min 54 sec	0.0779, 0.0789	0.0760, 0.0698	0.0770, 0.0744	0.0580, 0.0560
		0.001	81 min 41 sec	0.0088, 0.0095	0.0127, 0.0113	0.0107, 0.0104	0.0118, 0.0115
		0.0001	113 min 41 sec	0.0021, 0.0025	0.0078, 0.0070	0.0050, 0.0047	0.0105, 0.0097

### Experimental Results on Real Data

**Figure 4:**
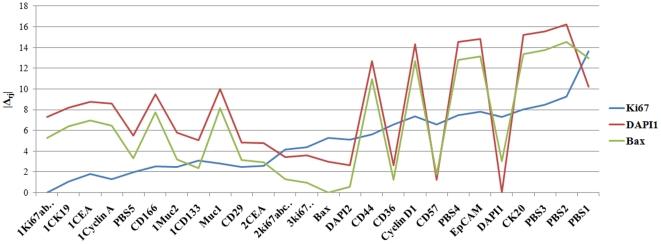
Magnitude of shift required to register different tag images to the corresponding reference image (tags along *x*-axis are arranged in increasing order of magnitude of shifts, *φ* calculated with respect to Ki67 as the RIMO image.)

We have run the proposed registration framework and the algorithm for selection of RIMO on a large number of TIS stacks. Here we report results of a TIS run on a cancerous colon tissue captured by the biologists S. Abouna and M. Khan in October 2010. The antibody tag library for the experiment consisted of tumor markers, stem cell markers, and proliferation markers. More details about this can be found in an earlier study [34]. First, we choose any arbitrary tag image (eg, DAPI) as a reference image 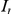 and calculate the transformations 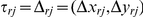 required to align all the images 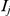, for *j = 1,2,…,N* with 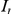. Using the results of registration, the RAMTaB gave Ki67 tag image as the RIMO. Registration results were also generated by arbitrarily choosing the Bax tag as reference. The results of registration using 3 reference images (DAPI and Bax selected arbitrarily, and Ki67 as RIMO) are shown in Figure 4 in the form of a plot of magnitude of shift required to register a tag image to the corresponding reference image. The plots clearly show that by using Ki67 as reference tag, the total amount of shift required to register the images is much smaller than by using the other two reference images. When Ki67 was used as reference image, there was only one tag for which magnitude of shift was found to be greater than 10, whereas, when DAPI1 or Bax were used as reference images, there were more than 8 images for which the magnitude of shift calculated was greater than 10. Since our goal is to minimize 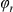, it is clear from Figure 4 that the RAMTaB framework has been successful in minimizing the magnitude of shifts.

**Figure 5:**
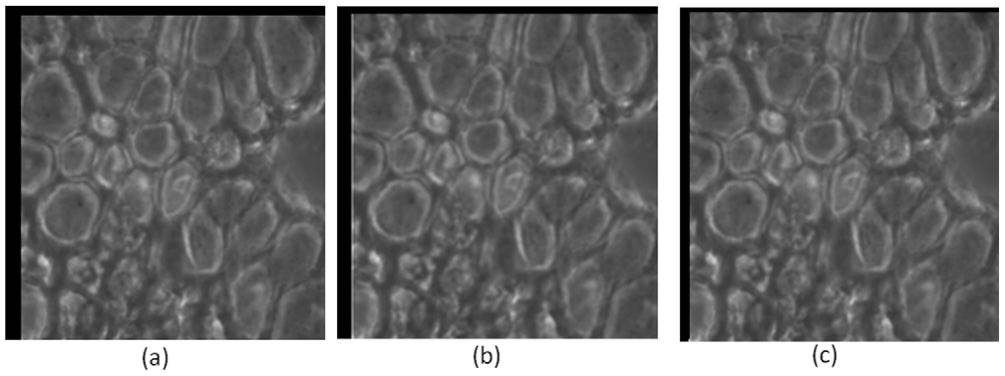
Phase contrast image for the CK20 tag registered to (a) DAPI1, (b) Bax and (c) Ki67 tag image; the black region on the top and the left of the image shows the amount of shift required to register the image to respective reference.

**Figure 6:**
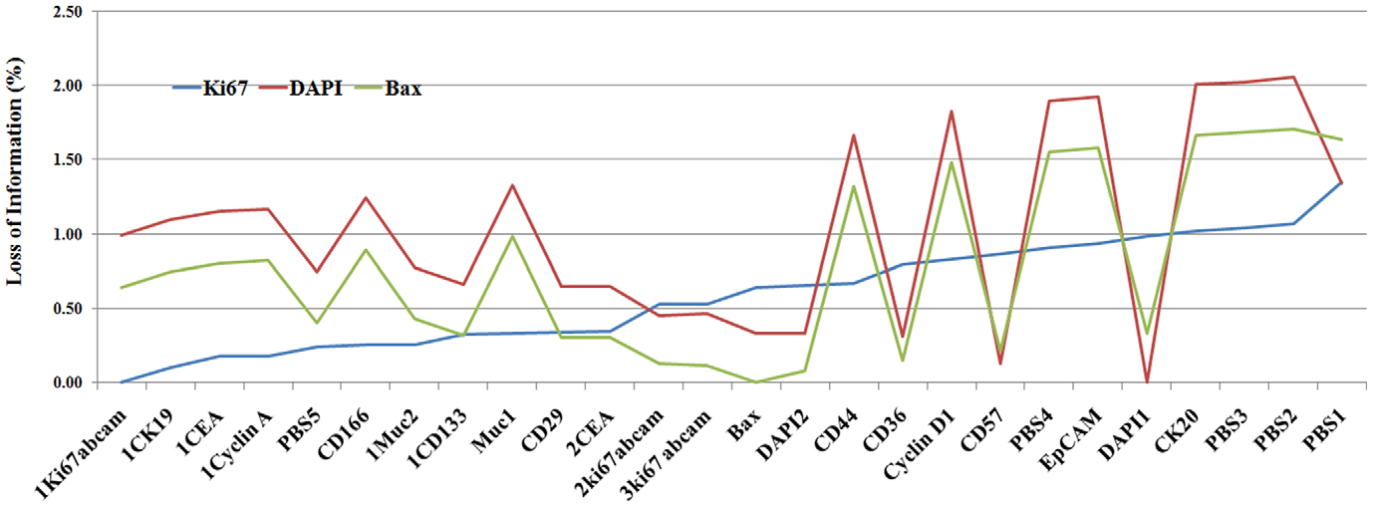
Percentage loss of information when registering using different channels as reference.

**Figure 7:**
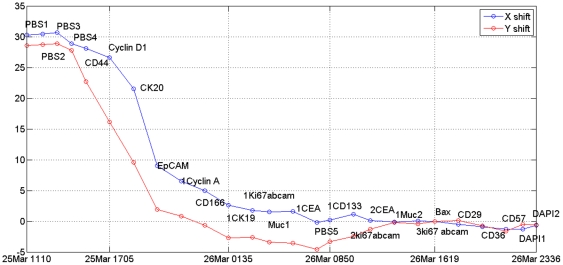
Shift, as estimated by RAMTaB, in both *x* and *y*-directions during one TIS run versus time. This indicates the TIS machine “settling down” to an equilibrium state as time passes, probably due to the temperature reaching a stable value.

Figure 5 shows the upper-left part of the phase contrast image for CK20 tag from the same image stack, after it has been aligned to the phase contrast images corresponding to DAPI1, Bax, and Ki67. The blank rows and columns (having zero intensity values) near the top-left corner of the image are due to the amount of shift which was required to align the image to the respective reference image. The number of blank pixels near the top left corner in this image is equal to the number of pixels lost at the bottom left corner of the image. It can be observed from this Figure that when the Ki67 image is used as a reference, 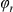 is minimized, once again showing in empirical terms that the proposed RAMTaB framework selects RIMO as reference for registration. Figure 6 shows the percentage loss of information when Ki67, DAPI and Bax were used as reference. Figure 7 shows the amount of translational shift calculated using the proposed RAMTaB framework for images acquired during a single TIS run plotted against time. In this particular instance, the amount of shift decreased as the TIS run progressed but in other cases, the trend may be different. This indicates that the TIS machine settles down to a stable state as the run continues.

As can be observed in Figure 7, the amount of misalignment varies from one tag image in the TIS image stack to another. Figure 1 show composite images formed by using CD57, CD166 and DAPI as R, G and B channels of a color image. The misaligned composite RGB color image using phase and fluorescence images are shown in part (a) and (b) respectively. The color fringes show the degree of misalignment between the tag images before registration. The aligned images are shown in part (c) and (d) of Figure 1.

Table 1 shows time consumed using different number *K* of the subimages and for different threshold specified for 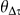 for a stack of 26 images with two visual fields on a 2.66 GHz Quad Core CPU. It was found empirically that 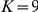, 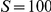, and 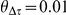 gave us a good compromise between the algorithm's runtime and the accuracy of registration. Using these parameters, our approach takes about 22 minutes and 15 seconds to register a stack of 26 tag images with two visual fields on a 2.66 GHz Quad-core CPU using non-optimized MATLAB® code running on a Linux platform. The MATLAB source code, 32-bit Windows executable, and a sample TIS stack can be downloaded from the project website. http://www2.warwick.ac.uk/fac/sci/dcs/research/combi/projects/bic/ramtab/.

## Discussion

A cell can be considered as an assembly of different molecules and proteins which interact together to define all cell functions. Most flourescence microscopy techniques are limited to up to ten fluorescent tags which can point to simultaneous localization of the corresponding biomolecules inside the cells of a tissue specimen [35]. The TIS system provides us with a platform to decode and locate hundreds of protein combinations at a given point in a cell. The TIS method uses a library of fluorescent tags to obtain phase-fluorescence pair images corresponding to each tag. Accurate alignment of tag images in a multi-tag fluorescence microscopy image stack is an essential pre-processing step prior to any analysis of protein co-expression. Unless this can be achieved, many important biological questions, such as cell classification and discovery of functional protein networks within a cell at different points in time, cannot be addressed. Here, we have presented an approach to select a reference image with maximal overlap. To the best of our knowledge, this problem has not been addressed in the literature before. The proposed framework determines sub-pixel shifts between phase contrast images in a multi-tag fluorescence image stack. Subsequently, these shifts can be used to register the fluorescence images to co-localize signals from different protein molecules or find molecular co-expression patterns for different biomolecules. Importantly, our system is highly effective on real as well as on synthetic data. It has been shown to be robust to luminance and contrast variations, yields a confidence value in the quality of alignment results, and removes the need for a biologist to eyeball all phase contrast images in the stacks to select an appropriate reference image. Our block-based registration algorithm ensures that the alignment is robust to any damage caused during sequential bleaching or washing to a small part of the tissue. On the synthetic data, the proposed framework gives almost perfect alignment, up to two decimal places sub-pixel accuracy for a selected set of parameters (see Table 1). The alignment accuracy can be increased using different set of values for *K*, *S*, and 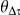, but the time required to register the stack also increases. On the real data, the selection of arbitrary image at the first step is very important. The image must be of a good enough quality or it may give misleading results.

Figure 1 shows composite RGB images obtained by using three different phase contrast and fluorescence images for CD57, CD166 and DAPI as red, green and blue channels, respectively. In Figure 1(a,b), phase contrast and fluorescence images from the original data set obtained after a TIS run are used as red, green and blue channels. The color fringes in Figure 1(a) show the degree of misalignment present between these three phase contrast images which should ideally be aligned to each other. We have aligned the images using the proposed algorithm and formed the composite as shown in Figure 1(c,d). If pixel intensities from all three phase contrast images are in agreement with each other, we should only see shades of grey in the composite RGB image. It can be seen in the alignment results of both the algorithms that the color fringes have been removed in Figure 1(c). We have calculated the root mean squared (RMS) difference between the red, green and blue channels, for the phase contrast images shown in Figure 1 to numerically illustrate the misalignment, using the equation below,
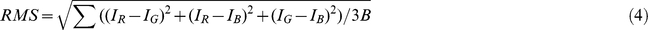
where *I_R_*, *I_G_*, and *I_B_* denote the red, green, and blue channel images, respectively, and *B* denotes the number of pixels in each of the channel images. For the images shown in Figure 1(a,c), the RMS difference was found to be 7.14 for the misaligned images (Figure 1(a)) and 2.98 for the registered images (Figure 1(c)). After registration, selection of the RIMO image is the next step. We have shown that we can collect maximum amount of data from the image stack after registration using the RIMO image. All the images are registered to this reference image using a novel shift metric. In a follow-up study [15], we have collected more TIS stacks of both normal and cancerous colon tissue from different patients. The RAMTaB framework has been successfully used to register several stacks and has been shown to be robust to brightness and contrast variations. However, there are some alignment difficulties with poor quality phase contrast images containing blur caused by changes in the plane at which camera sets its focus while taking the images. These changes in the focal plane are very rare. When they occur, they are probably due to mechanical problems with the shutter, or to minute particles contaminating the specimen, in which case the autofocus mechanism may focus on a particle rather than on the specimen, but there may be other factors of which we are not yet aware. Future work will address the issue of non-uniform focus in the image data.

## Materials and Methods

### Image Acquisition

The approval for this research has been granted by the Warwickshire Local Research Ethics Committee, Warwickshire, UK. The human tissue has been collected from operative samples at the George Eliot Hospital, Nuneaton, UK. Written patient consent was obtained to remove and use the tissue sections for research purposes before removing the tissue from the patient. After collection, tissues were immediately fixed in para-formaldehyde solution. After overnight cryo protection in sucrose solution, tissues were embedded in OCT blocks and stored frozen. Tissue sections were cut from each block and placed on coverslips. These sections were air-dried after incubating in ice-cold acetone. For TIS imaging, these tissues were incubated in sterile Phosphate Buffered Saline (PBS), in PBS containing normal goat serum, and then washed in PBS. See [34], [36] for more details. The images were acquired using the TIS machine installed at the University of Warwick. TIS has four main components: an epifluorescence microscope, a library of fluorescent tags (antibodies, lectins and DAPI), a robotic arm to handle the pipette, and a cooled CCD camera. More details about TIS can be found in [11], [36], [37]. We employed a library of 26 tags [38] consisting of a variety of cell specific markers, together with tumor and stem cell markers. Additionally, the nuclear marker DAPI were used and four PBS control tags. Tags were applied sequentially to the tissue section. An image is acquired before and then again after incubation with each fluorophore-conjugated antibody or other fluorescent dye, and washes to remove unbound tag. Each image is captured at 63× and has a spatial resolution of 1056×1027 pixels, where each pixel has a resolution of approximately 200 *nm*. Non-destructive photobleaching clears the fluorescence after each tag incubation once the image has been acquired. During the bleaching procedure, the sample is washed with PBS to minimize the background signal. The cycle of incubation, wash, image acquisition and photobleach is repeated then for another tag.

### The Proposed Framework

The proposed framework for multi-tag fluorescence image registration has three sequentially connected components in the following order: registration of all tag images in a stack to an arbitrarily chosen tag image as a reference image, selection of RIMO, and re-alignment of all images in the stack to RIMO. Below we describe the core registration algorithm based on mutual information used by the first and the third components. A side benefit of RAMTaB is that even if the arbitrarily chosen reference image is different from the RIMO, the core registration algorithm does not have to be executed again (See Section 5.3).

#### The Core Registration Algorithm

We employ a mutual information based framework [17] for registering one phase contrast image with another. Several other researchers have shown mutual information to be a good similarity measure for microscopic images [30], [39], [40]. Mutual information based on Hartley's entropy measure is defined as follows. Let 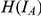 and 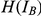 denote the entropies of 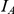 and 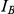, respectively, and let 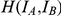 be the joint entropy of 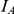 and 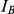. Then 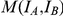, the mutual information between the two images 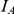 and 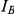 is defined by the formula
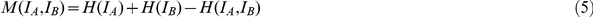
One approach to registering the two images is to maximize 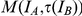, by varying 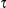 over some set of transformations. In our case, we vary 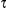 only over translations. Maximizing mutual information implies minimizing the joint entropy. Marginal and joint entropy can be calculated from the joint histogram, which is formed using the intensity values of the two images. When mutual information is high, the joint histogram is sharp and closely resembles a diagonal matrix. In a mutual information based registration framework, we transform the target image 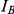 to match the reference image 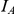 by searching for a transformation which maximizes the mutual information between the reference image and the transformed target image. Mathematically, this can be written as,
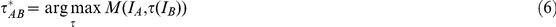
where 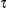 denotes the transformation between source and target images required to align them. The optimization is done using the *pattern search* method [41], [42]. At each step, the search algorithm creates a set of points called a *mesh* around the optimal point of the previous step. The pattern search finds a point that improves the objective function. If the algorithm fails to find such a point, it decreases the size of the mesh, otherwise it chooses the new point which has improved the objective function as the new optimal point and increases the size of the mesh in the next step. This search continues until 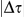 is less than a specified threshold 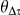 or the number of iterations reaches the maximum allowed number of iterations. In general, the transformation 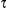 could consist of affine and perspective transformations. In our case, however, rotations and non-rigid transformations are not required, and therefore we are only concerned with horizontal and vertical movements between the target and reference images. Sub-pixel accuracy is achieved using bicubic interpolation [43] for sub-pixel shifts.

#### Measure of Confidence in the Registration Results

The method of registration described above is prone to get stuck in local maxima while optimizing for mutual information. There are several other problems. In the formula for mutual information, we need to get round the problem of the changing size of the intersection as 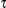 changes. It is also possible that no meaningful registration is possible. This would be the case if, for example, repeated washes during a TIS run were to tear the specimen, or if new extraneous material were to float into the visual field.

**Figure 8:**
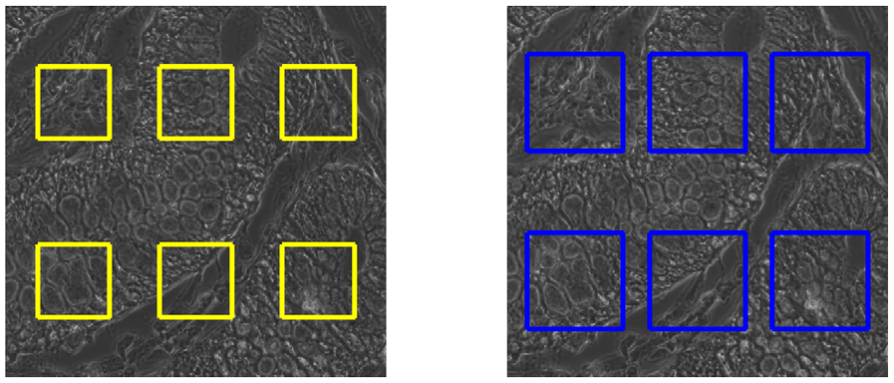
A total of *K* sub images are extracted from different locations in the reference image *I_r_* (left) and optimal translations τ*^k^_rj_* for *k* ∈ {1,2, …,*K*} are calculated between the *k*-th subimage in *I_r_* and its corresponding sub image in the target image *I_j_* (right) using a search neighbourhood. Based on these individual transformations, the overall rigid transformation τ*_rj_* required to register the images is calculated.

To obtain more reliable registration results capable of detecting such failures, we select *K* disjoint square subimages from the reference, and *K* somewhat larger disjoint square subimages from the target image, as in Figure 8. Each such square in the target image corresponds to exactly one square in the reference image, and the corresponding squares have centers at the same positions in target and reference images.

We register each of these square subimages of the reference image within the corresponding larger square in the target image. More precisely, we find *K* translations 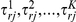, where 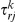 is the optimal translation registering the *k*-th smaller square in the reference image within the *k*th larger square in the target image. Now 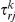 is the translation by a certain 2-dimensional vector, which we denote by
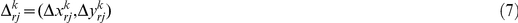
We then calculate the pairwise Euclidean distances *d_kl_* between 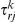 and 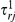, for *k, l* = 1,2,…,*K*. If, for fixed *k* and for all *l*, the value of *d_kl_* is greater than *ω* pixels (where *ω* is some previously chosen number), we mark *k* as an outlier and the user can be warned that this has occurred, making visual inspection possible. If for fixed *r* and *j*, fewer than *k*/2 translations 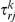 are marked as outliers, their *k* indices are added to the outlier set 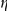, to be excluded from any further calculation. This ensures that if a small number of registrations (fewer than *k*/2) disagree with the majority, they are safely removed from the computation. We found *ω* = 1 suited for our experiments.

A major benefit of registering with subimages is that one can easily compute a measure of confidence in the registration results in terms of the standard deviation of the shifts:
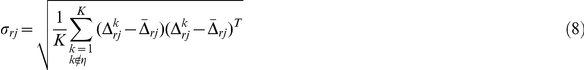
where
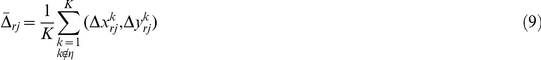
The standard deviation 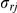 can be used as a measure of confidence in the registration results. If this value is larger than a specified threshold, then the registration process is performed again using a slightly different set of square subimages. If the confidence value is again larger than the specified threshold, we flag the target image as a potentially bad quality image or an image that cannot be registered well. If the standard deviation is below the specified threshold for satisfactory registration, the translation 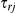 is computed as the average of all non-outlier local transformations between subimages as given in equation (9).

Note from Table 1 how the time taken by the pattern search algorithm depends crucially on the accuracy 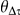 demanded. Moreover, the accuracy of the final result cannot sensibly be better than the size of the standard deviation, as the standard deviation is a good estimate of the intrinsic error in the measurement. This indicates a possible speed-up in our program by interleaving calls to the pattern searches with computations of the standard deviation, stopping when the standard deviation indicates that one has reached the limits of what one can reasonably expect for the precision of the registration translation required for that particular image.

### Selection of Reference Image with Maximal Overlap (RIMO)

In this section, we utilize these transformations between all images 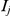, for *j* = 1,2,…,*N*, and 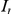 in order to select the RIMO maximizing the total overlap between the aligned images as shown in Figure 2.

#### Registration Graphs

**Figure 9:**
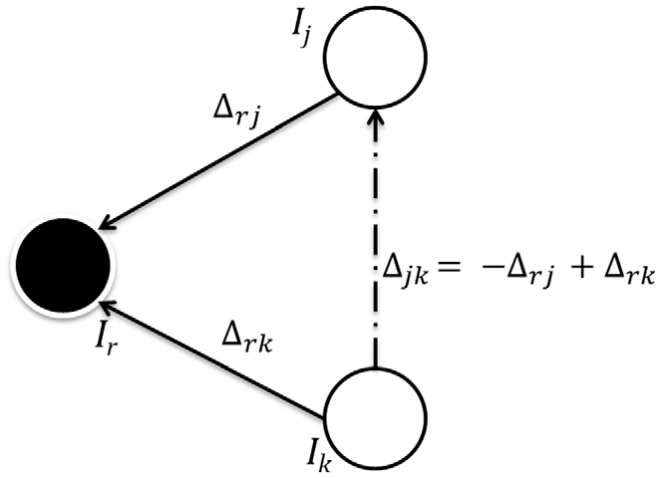
Finding the shift Δ*_jk_* between the images *I_j_* and *I_k_*, using the previously calculated shifts Δ*_rj_* and Δ*_rk_* with image *I_r_*. This is similar to vector diagrams where Δ*_jk_* is the resultant vector.

**Figure 10:**
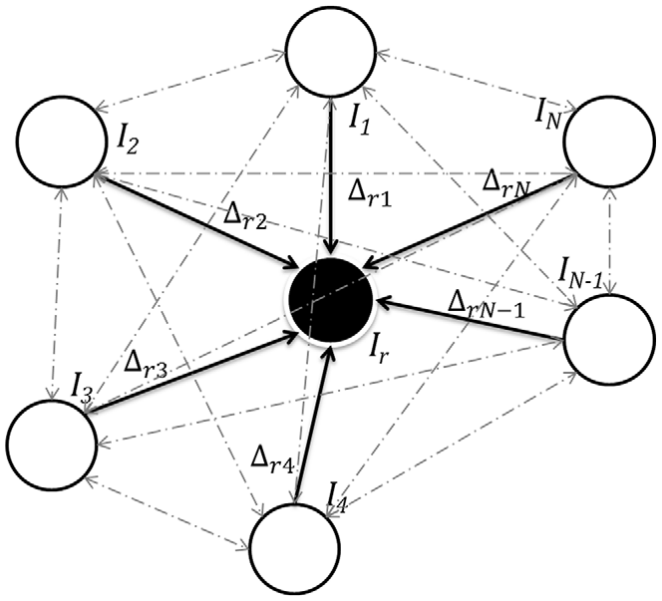
Registration graph showing shifts calculated between *I_r_* and all the other tag images in the dataset I = {*I_j_*}, *j* = 1,2,…,*N*. Nodes in the graph represent multi-tag images in an image stack **I**, solid edges represent transformations with respect to *I_r_* as described in Section 5.2.1, and dashed edges represent transformations that can be determined using this graph as shown and described in Figure 9.

First, we choose any arbitrary image 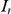 having good enough quality as a reference image and calculate the transformations 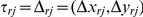 required to align all the images 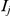, for *j = 1,2,…,N* with 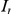. Once these shifts have been calculated, we can compute the pairwise transformations 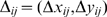 between any two images 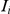 and 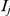 in the dataset **I**, as shown in Figure 9. The pairwise transformations can then be arranged in the form of two *inter-tag shift matrices* as given below.
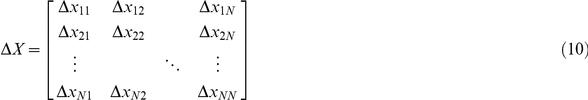


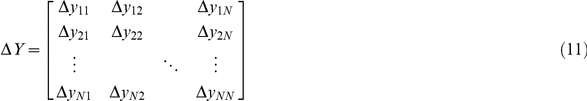


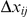
 and 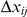 represent shifts along *x*-direction and *y*-direction of image 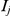 with 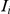 as the reference image. The above matrices can also be represented in the form of a registration graph, as shown in Figure 10. The registration graph can then be used to find shifts between any pair of images in the set **I**, as shown in Figure 9. We can now complete the matrices 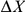 and 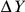 with the help of the equation obtained from the registration graph,
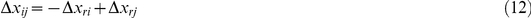


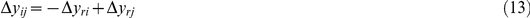
The above equations give shifts required by any image 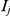 considering 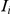 as the reference image. Since the resultant matrix is skew-symmetric, we can first compute the upper diagonal matrix and then compute the lower diagonal by just flipping the matrix about the diagonal with a negative sign, to reduce the amount of computation. The total number of registrations performed for *N* tag images is *N*−1, producing 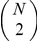 shift values using equations (12) and (13).

#### The Objective Function

We wish to compute the value of *r* that maximizes 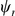, as defined in equation (1). This could be done by direct computation. However, we will show that this expression is also given in terms of a certain metric that we will define. The metric will be a special case of a very general metric coming from a measure in the sense of mathematical Measure Theory.

#### The Shift Metric

We now discuss the metric associated with our objective function. For this discussion, we need a collection S of subsets of a fixed set 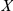 and a function *μ* : S→[0, ∞) satisfying the conditions for S to be a semiring, and for *μ* to be a measure. The only examples that we will use in this paper are:

- S is the set of all finite subsets of the plane, and *μ*(S) is equal to the number of elements in S (counting measure). In fact, we will restrict our attention to the situation where the plane is divided into a fixed set of pixels, and each point of S is at the center of some pixel. Then *μ*(S) is just a count of pixels.
- S is the set of all rectangles in the plane, not necessarily with vertices at integer points, and *μ*(S) is the usual area of the rectangle. We will assume that the plane is divided into square pixels of height and width one, so that *μ*(p) = 1 for any pixel *p*.

The symmetric difference of two subsets 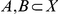
is defined as,
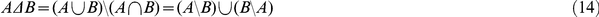



#### Lemma 1

For any 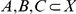,
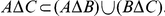



### Proof

Suppose 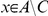. Then 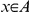 and 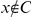. If 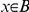, then 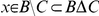. If 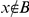, then 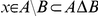. This shows that 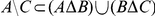. Now suppose 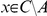. Then 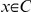 and 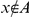. If 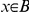, then 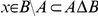. If 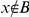, then 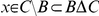. This shows that 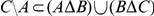.

Thus 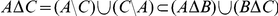.

Recall that a *pseudometric d* satisfies the same axioms as a metric, except that *d*(*x*,*y*) = 0 does not necessarily imply *x* = *y*.

#### Theorem 1

Let μ be a measure on X, and let □ be the set of subsets of finite measure. Then we obtain a pseudometric *d* on □ by defining 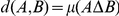. This is a metric if *μ* has the property that *μ*(*A*) = 0 implies *A* = ∅, the empty set.

### Proof

For all 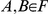, we have 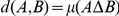
≥0. Since 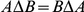, we see that 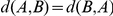. For any 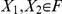, we know that 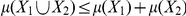, with equality when 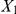 and 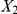 are disjoint. (This is true for any measure, and can be directly checked for our two examples of counting measure and area.) It follows from Lemma 1 and this inequality that
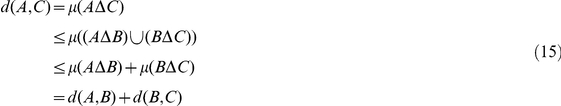
This shows that *d* is a *pseudometric*. If, in addition, 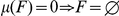, then
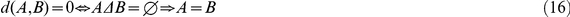
which is the final axiom needed in order to show that *d* is a metric.

**Figure 11:**
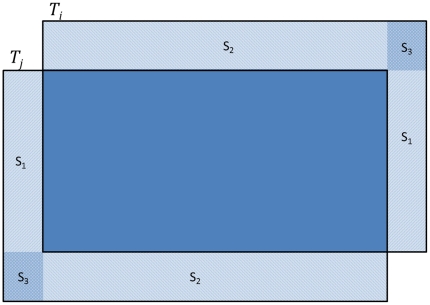
Calculating the area occupied by the non-overlapping region.

Let us apply this result to the example of Figure 11. We fix a reference image *I_r_*, and target image *I_j_*. Let 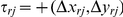, for 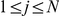. Then 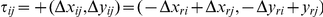 as shown by the registration graphs in Figure 9 and Figure 10. Using the above metric *d* on subsets of the plane, we define ∥τ_ij_∥*_d_* = *d*(*C_i_, C_j_*), though we caution that it is not a norm on the set of translations.

#### Lemma 2

Let *d* be the metric that arises from Theorem 1, applied to one of our two examples. Recall that each *I_j_* has height *h* and width *w* for all *j* = *1*,*2*, …, *N*. Then, for 1≤*i*, *j*≤*N*, we have
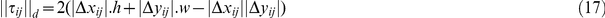
provided that 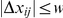 and 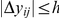. If 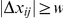 or 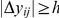 then 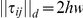. This is the area lost by aligning *I_j_* with *I_i_*.

### Proof

If 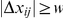 or 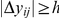 then 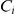 and 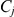 do not intersect, and so 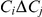 is the disjoint union of 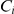 and 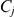, and this has area 2*hw*. Otherwise, the situation will be similar to that shown in Figure 11. From this figure, we see that the area of 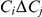, which is the area lost, is given by
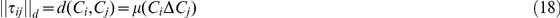
The region 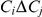 consists of two congruent components, each comprising three sub-rectangles *S*_1_, *S*_2_ and *S*_3_, meeting only along their edges, with *S*_3_ occupying the corner position. We calculate 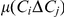 as follows:
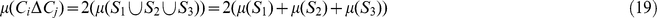
From Figure 11,
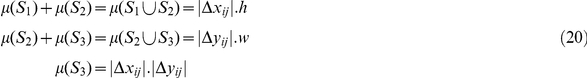
It follows that
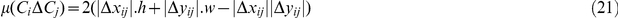
In practice though, 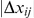 is typically much smaller than *w* and 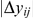 is much smaller than *h*. Moreover, the value at which the objective function is optimized is unchanged if the objective function is multiplied by a constant. As a result, the third term in the sum can be ignored, and a good approximation to the exact answer is a scaled version of the *l*_1_-metric, given by
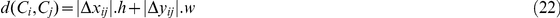
and this value can be used to specify the objective function. We can now revisit the objective function
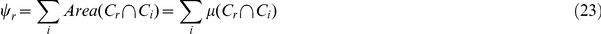
Note that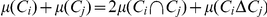. If *C_i_* and *C_j_* move, while keeping each of 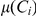 and 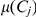 constant, then 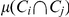 increases as 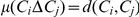 decreases. That is, the larger the area in common between two images, the smaller will be the distance between them. It follows that maximizing 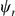 is equivalent to minimizing,
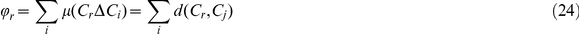
as *r* varies. Minimizing the above objective function gives r* the index of the RIMO image.

#### Using the RIMO

Once we have computed all the shifts, found the RIMO and its distance to each of the after tag images, we realign all the tag images with reference to the RIMO. Furthermore, we can also identify which of the fluorescence protein images it might be best to ignore, if for some reason it is advisable to ignore one or more images. Of course, one will often want to ignore fluorescence images of poor quality. But it may also be advisable to eliminate, at least temporarily, images that are distant from the RIMO (using the distance function defined above).

## Supporting Information

Appendix S1 **Various different ways in which the alignment results can be used for a follow-up analysis.**
(DOC)
