# Supplementary material for: RAMTaB: Robust Alignment of Multi-Tag Bioimages
Source: PLoS One. 2012 Feb 8;7(2):e30894. doi: 10.1371/journal.pone.0030894 (PMC3280195; doi:10.1371/journal.pone.0030894)
Supplement: Appendix S1 — Various different ways in which the alignment results can be used for a follow-up analysis. (DOC) [file pone.0030894.s001.doc]

## Appendix S1

## Supporting Information: Various different ways in which the alignment results can be used for a follow-up analysis

There may be several ways in which the registration results can be used for a follow-up analysis. Here we consider four possible options. A first option is that we select only those co-ordinates where all the signals overlap as shown by $C_{\cap}$in Figure 2. Note that we use the word *coordinates* instead of *pixels* because we wish to allow alignment transformations $\tau_{rj}$with sub-pixel accuracy. We consider all the aligned images in a universal co-ordinate system and let $C_{j}$denote the set of co-ordinates of$T_{rj}$. We define the set $C_{\cap}$, shown as the yellow shaded area in Figure 2, as the set of co-ordinates of the overlapping region as follows,

$$C_{\cap}=\bigcap_{j=1}^{N} C_{j}$$

A major limitation of using $C_{\cap}$ is that this set may be very small if we have a few images that are far from the center. We risk losing a large amount of signal in the non-overlapping region, when we can benefit from this signal at the cost of eliminating from consideration only a few of our images.

A second option is that we select signals from all the aligned images and form a larger mosaiced image as shown by the green dashed line in Figure 2. Let $C_{0}$be the set of co-ordinates of empty spaces in the larger mosaiced image, and let $C_{\cup}$be the set of co-ordinates of all the aligned images in the dataset as shown by shaded region with different colors in Figure 2. Let $C$be the set of co-ordinates of this larger rectangular image with dotted boundary. Then

$$C=C_{0}\cup C_{\cup}$$

where

$$C_{\cup}=\bigcup_{j=1}^{N} C_{j}$$

In this case, there may be a substantial region covered by only a few images, or even by no image at all, as in $C_{0}$, rendering the post processing of those elements potentially meaningless.

A third option is to find, for a particular value of *r*, which *r* images should be omitted in order that the intersection of the remaining images should have a maximal area. We will address this question in a future paper.

The fourth option is explained and discussed in the Section 1 of this paper.
